# Advances in the use of stem cell transplants in the treatment of multiple sclerosis

**DOI:** 10.1007/s00415-021-10927-6

**Published:** 2022-01-04

**Authors:** Rachel Thomas, Ray Wynford-Thomas, Neil P. Robertson

**Affiliations:** 1 Department of Neurology, University Hospital of Wales, Heath Park, Cardiff, CF14 4XN UK; 2 Department of Neurology, Division of Psychological Medicine and Clinical Neuroscience, Cardiff University, University Hospital of Wales, Heath Park, Cardiff, CF14 4XN UK

## Introduction

Since the first bone marrow transplant was performed in the 1950s, stem cell-based therapies have offered the potential to treat many chronic, degenerative conditions, and multiple sclerosis (MS) is no exception. Over the last decade, autologous haematopoietic stem cell transplantation (AHSCT), which aims to ‘reset’ the immune system, has been shown to have efficacy in reducing disease activity and disability progression in relapsing remitting (RRMS), leading to AHSCT becoming a viable therapeutic option for those with MS refractory to disease-modifying therapies (DMTs). This procedure however can be associated with significant morbidity, as it requires aggressive immunosuppression pre-transplantation. More recently, autologous mesenchymal stem cell transplantation (MSCT) has moved into the spotlight. MSCs are multi-potent, non-haematopoietic stromal cells, found mainly in the bone marrow. Pre-clinical trials have shown that they fulfil a potent immunomodulatory role and may have neurotrophic effects. Furthermore, in contrast to AHSCT, there is no requirement for pre-transplantation immunosuppression. However, the evidence of its efficacy in reducing MS disease progression has been limited. Three recently published phase II clinical trials investigating the effects of stem cell transplantation (AHSCT and MSCT) in MS are discussed below.

## Safety, tolerability, and activity of mesenchymal stem cells versus placebo in multiple sclerosis (MESEMS): a phase 2, randomised, double-blind crossover trial

The MESEMS multi-centre, crossover study aimed to establish the safety and activity of autologous intravenous (IV) MSCT in patients with active MS that had failed at least one DMT. 144 patients (65% RRMS; 23% secondary progressive MS (SPMS); 12% primary progressive MS (PPMS)) were randomised into two patient groups: the first (‘early MSC group’) received a single IV dose of autologous bone marrow-derived MSCs in week 1, followed by a placebo infusion at week 24; the second (‘delayed MSC group’) received a placebo infusion in week 1 followed by the single MSC infusion at week 24. No significant difference in the frequency of adverse events was noted between the patient groups. During the first 6 months of the study, 51% of patients in the early MSC group and 56% of patients in the delayed MSC group, experienced at least one adverse event. Between weeks 24 and 48, 59% of patients in the early MSC group and 59% of patients in the delayed MSC group reported at least one adverse event. The most commonly reported adverse events were infections/infestations, only one of which was felt to be likely related to treatment and occurred in the delayed MSC group between 24–48 weeks. Activity was evaluated by the total number of gadolinium-enhancing lesions (GELs) at week 24, and no statistically significant difference was observed between groups. The total number of GELs per scan over 24 weeks, adjusted for the baseline number of GELs, was 1.16 for the early MSC group and 1.24 for the delayed MSC group (estimated RR 0.94, 95% CI 0.58–1.50; *p* = 0.78).

***Comment: ***This is one of the largest studies analysing the effects of a single IV MSC transplantation on MS activity. Importantly, it found no safety issues compared to placebo. However, it also found no statistical difference in the reduction of MS activity. There remains wide scope to conduct further research into MSC use in the treatment of MS, including utilisation of alternatively derived MSCs and variable delivery methodologies, as well as the ever-thorny issue of identifying more reliable and meaningful outcome measures for MS.

*Uccelli A et al. (2021) Lancet Neurol. 20(11):917–929*

## Beneficial effects of autologous mesenchymal stem cell transplantation in active progressive multiple sclerosis

This phase II, randomised, double-blinded, crossover study aimed to establish the safety and efficacy of both intrathecal (IT) and IV MSC transplantation, in active or worsening progressive MS, in those who had failed at least one DMT. 48 patients (41 SPMS; 7 PPMS) were divided into three groups. Each group underwent two cycles of treatment. During the first treatment cycle, group 1 received a dose of MSC-IT, group 2 received a dose of MSC-IV and group 3 received placebo only. After 6 months, the groups were crossed over. Half of groups 1 and 2 received a second dose of their original treatment (either MSC-IV or MSC-IT), while the other half received placebo. Group 3 received either MSC-IT or MSC-IV. Blinding was achieved using sham IV or IT injections.

There was no significant difference in adverse events between patient groups (headache was the most common) and no serious adverse event was thought to be related to MSC treatment. The primary endpoint used to evaluate clinical efficacy was the percentage of patients who experienced treatment failure, measured by an increase in EDSS score of ≥ 1 point for patients with baseline EDSS values of ≤ 5, and of ≥ 0.5 for baseline EDSS > 5.0. EDSS was re-evaluated at the end of each treatment cycle (6 and 12 months). Treatment failure in patients treated with MSC-IT (6.7%) and MSC-IV (9.7%) was significantly lower than that compared to sham treatment (41.9%) (*p* = 0.0003 between MSC-IT and sham treatment; *p* = 0.0008 between MSC-IV and sham treatment). There was no statistically significant difference in treatment failure between the MSC-IV and MSC-IT groups. MSC-IT appeared to be superior to MSC-IV with regard to some secondary endpoints, such as the nine-hole peg test and PASAT cognitive test, and a repeated dose of MSC-IT at month 6 was reported to significantly boost the effects observed during the first cycle of treatment. There was no significant difference in the number of GELs between patient groups. Since 30 out of the 48 patients included had disease activity, the authors comment that MSC transplantation benefits predominantly active progressive MS.

***Comment: ***As in the MESEMS study, this study provides additional evidence to support the safety of MSC transplantation in MS. Furthermore, it provides evidence that MSC-IT/IV may be used to reduce disease progression in (predominantly active) progressive MS patients. Limitations of this study include the relatively small sample size, short duration and that the crossover design may have introduced a ‘carry over’ effect from initial treatment. In addition, although most treatments used pre-transplantation should not have had an ongoing effect on disease activity, since there was a washout duration of 3–6 months, one patient had received rituximab, with B cell depletion effects of 12 months or more. Future research studies using larger cohort numbers will be useful to further investigate the effects of MSC delivery method and repeated treatments on outcomes.

*Petrou P et al. (2020) Brain. 143(12):3574–3588*

## Prospective phase II clinical trial of autologous haematopoietic stem cell transplant for treatment refractory multiple sclerosis

In this single-centre, phase II study, 35 patients with RR (*n* = 20) or SPMS (*n* = 15), who showed clinical or radiological evidence of disease activity despite treatment with DMTs, were treated with BEAM (carmustine, etoposide, cytarabine, melphalan) and antithymocyte globulin chemotherapy, and then received an IV AHSCT. The median follow-up was 3 years and the primary outcome was event-free survival (EFS), defined by no EDSS progression, no clinically active disease and no radiologically active disease on MRI following a re-baseline scan at 6 months.

The estimated EFS probability for the whole cohort at 3 years was 60% (*n* = 14, 95% CI 40–75%). For the RRMS cohort only (who had failed a median of 4 DMTs), the EFS probability at 3 years was 70% (*n* = 8, 95% CI 41–87%). 88% of these patients had no progression of their EDSS scores at 3 years (95% CI 60–97%). The study found a sustained improvement in EDSS over 3 years, predominantly in those with RRMS. 83% of patients remained free of new or enlarging T2 lesions and 96% free of gadolinium enhancing lesions on last follow-up MRI (median time of 29 months). The survival rate was 100% and, despite the majority of patients (66%) having been treated with natalizumab previously (median time from last natalizumab treatment to AHSCT of 10 months) and 79% of patients having a positive JC virus, there were no cases of PML at a median of 3 years follow-up. 55 serious adverse events were reported and included infection and thrombosis, amongst others. Other side effects included mucositis, nausea, alopecia and the need for red cell/platelet transfusions.

***Comment***: Despite the absence of a placebo/DMT control arm preventing direct comparison with AHSCT, this study supports evidence that AHSCT is a viable therapeutic option for DMT-refractory RRMS. Furthermore, an important negative finding is the suggestion that those with SPMS may have a less favourable response, even if active. Despite the evidence for its efficacy in RRMS, it is imperative that the morbidity associated with AHSCT is considered carefully when choosing treatments; future trials with a larger sample size, comparing AHSCT to placebo/DMT, will play a vital role in informing both patients and treating physicians.

*Moore J et al. (2019) Journal of Neurology, Neurosurgery & Psychiatry. 90(5):514–521.*

